# Pollinator declines: reconciling scales and implications for ecosystem services

**DOI:** 10.12688/f1000research.2-146.v1

**Published:** 2013-07-02

**Authors:** Ignasi Bartomeus, Rachael Winfree

**Affiliations:** 1 Department of Ecology, Swedish University of Agricultural Sciences, Uppsala, SE-75007, Sweden; 2 Department of Ecology, Evolution and Natural Resources, Rutgers University, New Brunswick NJ, 08901, USA

## Abstract

Despite the widespread concern about the fate of pollinators and the ecosystem services they deliver, we still have surprisingly scarce scientific data on the magnitude of pollinator declines and its actual contribution to crop pollination and food security. We use recently published data from northeastern North America to show that studies at both the local and regional scales are needed to understand pollinator declines, and that species-specific responses to global change are broadly consistent across scales. Second, we show that bee species that are currently delivering most of the ecosystem services (i.e. crop pollination) are not among the species showing declining trends, but rather appear to thrive in human-dominated landscapes.

## Main text

There is widespread concern regarding the fate of pollinators and the ecosystem services they deliver ^1^. However, the information we have is still limited and at times appears contradictory. Four recent articles, three from *Science* and one from *PNAS*, highlight this point ^2– 5^. Burkle *et al.* ^2^ show that 50% of the bee species in one locality in the Midwestern USA became locally extinct during the last century, which in combination with recent evidence that wild pollinators are critical to global crop pollination ^3^, has led some to conclude that we might face an imminent collapse of crop pollination ^4^. In contrast, Bartomeus *et al.* ^5^ explored bee declines over a similar time scale but at a regional scale (the northeastern USA) and reported only a 15%, non-significant decline in bee species richness. Here we present new analyses that help to reconcile this apparent contradiction in the magnitude of bee declines, while also suggesting that any effects on crop pollination might be less than previously thought.

**Figure 1.**
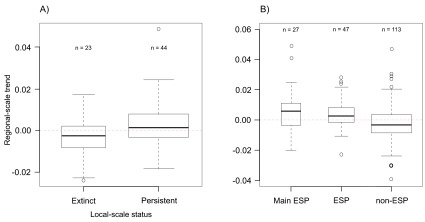
Trend in bee species' relative abundance in northeastern North American calculated over the period 1870–2011. **A**) For species that either became locally extinct or persisted in Carleville, Illinois. **B**) For species that either are not ecosystem-service providers to crops (non-ESP), are at least occasionally ecosystem-service providers to crops (ESP), or are among the species cumulatively responsible for 90% of the pollinator visitation to at least one crop (main ESP). Regional data from Bartomeus *et al.* ^5^, local data from Burkle *et al.* ^2^ and crop pollinator data from Garibaldi *et al.* ^3^.

First, we used the 67 bee species included in both the regional-scale ^5^ and the local-scale ^2^ analyses (see data file below) to show that the two studies in fact found broadly consistent results: the locally extinct species of Burkle ^2^ tend to be declining regionally, whereas the locally persistent species tend to be increasing regionally ( Figure 1A, ANOVA: F = 5.89, df = 1,65, P = 0.01). Second, we used data from Garibaldi *et al.* ^3^ on the bee species that provide ecosystem services to four crops in the region covered by Bartomeus *et al.* ^5^ to show that these ecosystem service providers tend to have increasing population trends compared to non-ecosystem service providers ( Figure 1B, F = 7.12, df = 2,184, P = 0.001). All analyses were conducted in R ^6^.

Northeastern North American bee species information on population trends and ecosystem services delivered Regional-scale trend from Bartomeus et al. (2013) in relative abundance calculated over the period 1870-2011. Local-scale status from Burkle et al. (2013) shows extinction or persistence in Carleville, Illinois. Ecosystem Service Provider is classified in non-ESP (not ecosystem-service providers to the studied crops), ESP (occasionally ecosystem-service providers to the studied crops), and main ESP (among the species cumulatively responsible for 90% of the pollinator visitation to at least one of the studied crop) according to data from Garibaldi et al. (2013). See main article for references.

Thus, our analyses demonstrate that, as one would expect, local-scale extinctions do not imply regional-scale extinctions; and that bee species that are important crop pollinators are less likely to be declining at the regional scale. It is important to remember that all bee species may well be crucial to providing ecosystem functions in natural systems and therefore merit conservation attention.
