# Supplementary material for: Pollinator declines: reconciling scales and implications for ecosystem services
Source: F1000Res. 2013 Jul 2;2:146. [Version 1] doi: 10.12688/f1000research.2-146.v1 (PMC3892917; doi:10.12688/f1000research.2-146.v1)
Supplement: Peer review history

# Peer review: Referee response for version 1

**Published:** 2013-07-03

**Authors:** Ryan Chisholm

**Affiliations:** 1 Department of Biological Science, National University of Singapore, Singapore, 119077, Singapore

This is a straightforward analysis and the interpretations are justified. I have two minor comments:

- Figure 1B shows that ecosystem-service-providing (ESP) bee species tend to be increasing whereas non-ESP species tend to be decreasing. But how is ESP measured? Is there any possibility that this result could be artefact, in that declining species are now rare and that therefore their ESP behaviour is less likely to be recorded? I would like to see this issue addressed briefly.
- The final sentence “It is important to remember that all bee species may well be crucial to providing ecosystem functions in natural systems and therefore merit conservation attention.” seems like a non-sequitur, because the authors have just finished talking about how important pollinators are less likely to be declining. Presumably the authors mean that we have incomplete information and that it would be risky to assume that apparent non-pollinators are genuinely playing no useful ecosystem-service role. This could be expressed better.

I have read this submission. I believe that I have an appropriate level of expertise to confirm that it is of an acceptable scientific standard.

# Response

**Published:** 2013-07-10

**Authors:** Ignasi Bartomeus

**Affiliations:** Department of Entomology, Rutgers University, USA

Regarding your first comment, ESP is measured as any bee species visiting flowers of at least one of four main crops the study area, based on data we collected in the recent period (2004-2011) and published in Garibaldi *et al., *2013. Thus the reviewer is correct that there could have been species that were ESP in the past but, due to population decline, were not recorded as ESP in our recent surveys, and our results should be interpreted in this light. However, we believe that our finding, that ESP are declining less than other species, has generality for two reasons. 1) We report in Bartomeus *et al.* 2013 that most of the bee species that have been declining over the past 140 years in our study region are still frequently recorded, hence our measure of ESP as "any species observed visiting crops at least once" should capture declining species as well if they commonly visit crops  2) Even the current ESP are a subset of the historical ESP, Garibaldi *et al., *2013 shows that the under this current situation, wild pollinators enhance fruit set, suggesting that the resilience of the current ESP to global change may buffer the effects of a pollinator decline. Lastly, on a priori grounds it would not be surprising if ESP were more robust to land use change (a primary form of global change in our study region) than non-ESP because by definition species found pollinating crops are able to persist in agricultural areas.

Regarding your second question, it is important to note that the last sentence refers to "natural systems." While our data and analyses refer to simplified crop systems, where a few dominant pollinators are responsible for most of the function delivered to a single plant species (the crop), natural systems are far more complex and high levels of bee diversity are likely to be necessary to provide function to the full community. Hence our results should not be extrapolated to other, non-agricultural systems. 

Ignasi Bartomeus & Rachael Winfree

# Peer review: Referee response for version 1

**Published:** 2013-07-03

**Authors:** Gary Luck

**Affiliations:** 1 Department of Wildlife Ecology and Management, Charles Sturt University, Albury, Australia

This short article comes to the important conclusion that while declines in pollinator [bee] abundance at local and regional scales are generally consistent, these declines are not occurring among those species responsible for delivering the majority of pollination services to particular crops. This insight makes a valuable contribution to the recent debate on the implications of pollinator declines for food production and will hopefully spur more studies that look closely at the relative contribution of different species to delivering pollination services across different crop types, and how the abundance of these species has changed over time.

I have read this submission. I believe that I have an appropriate level of expertise to confirm that it is of an acceptable scientific standard.
